# Association of *NOS1* gene polymorphisms with cerebral palsy in a Han Chinese population: a case-control study

**DOI:** 10.1186/s12920-018-0374-6

**Published:** 2018-06-25

**Authors:** Ting Yu, Lei Xia, Dan Bi, Yangong Wang, Qing Shang, Dengna Zhu, Juan Song, Yong Wang, Xiaoyang Wang, Changlian Zhu, Qinghe Xing

**Affiliations:** 1 Children’s Hospital and Institutes of Biomedical Sciences, Fudan University, Wanyuan Road 399, Minhang District, Shanghai, 201102 China; 2 Henan Key Laboratory of Child Brain Injury, Third Affiliated Hospital of Zhengzhou University, Kangfuqian Street 7, Zhengzhou, 450052 China; 3 Department of Pediatrics, Zhengzhou Children’s Hospital, Zhengzhou, 450053 China; 4 Perinatal Center, Institute of Neuroscience and Physiology, University of Gothenburg, Gothenburg, 40530 Sweden; 5 Center for Brain Repair and Rehabilitation, Department of Clinical Neuroscience, Sahlgrenska Academy, University of Gothenburg, Gothenburg, 40530 Sweden; 6 Shanghai Center for Women and Children’s Health, Shanghai, 200062 China

**Keywords:** Cerebral palsy, Nitric oxide synthase 1, Single nucleotide polymorphism, Association analysis

## Abstract

**Background:**

Cerebral palsy (CP) is the leading cause of motor disability in children; however, its pathogenesis is unknown in most cases. Growing evidence suggests that Nitric oxide synthase 1 (NOS1) is involved in neural development and neurologic diseases. The purpose of this study was to determine whether genetic variants of *NOS1* contribute to CP susceptibility in a Han Chinese population.

**Methods:**

A case-control study involving 652 CP patients and 636 healthy controls was conducted. Six SNPs in the *NOS1* gene (rs3782219, rs6490121, rs2293054, rs10774909, rs3741475, and rs2682826) were selected, and the MassARRAY typing technique was applied for genotyping. Data analysis was conducted using SHEsis online software, and multiple test corrections were performed using SNPSpD online software.

**Results:**

There were no significant differences in genotype and allele frequencies between patients and controls for the SNPs except rs6490121, which deviated from Hardy-Weinberg equilibrium and was excluded from further analyses. Subgroup analysis revealed differences in genotype frequencies between the CP with neonatal encephalopathy group (CP + NE) and control group for rs10774909, rs3741475, and rs2682826 (after SNPSpD correction, *p* = 0.004, 0.012, and 0.002, respectively). The T allele of *NOS1* SNP rs3782219 was negatively associated with spastic quadriplegia (OR = 0.742, 95% CI = 0.600–0.918, after SNPSpD correction, *p* = 0.023). There were no differences in allele or genotype frequencies between CP subgroups and controls for the other genetic polymorphisms.

**Conclusions:**

*NOS1* is associated with CP + NE and spastic quadriplegia, suggesting that NOS1 is likely involved in the pathogenesis of CP and that it is a potential therapeutic target for treatment of cerebral injury.

**Electronic supplementary material:**

The online version of this article (10.1186/s12920-018-0374-6) contains supplementary material, which is available to authorized users.

## Background

Cerebral palsy (CP), the most common cause of motor disability in childhood, is a group of permanent movement and posture disorders attributed to non-progressive abnormalities in the immature brain. Patients with CP often exhibit secondary musculoskeletal problems, epilepsy, and disturbances of sensation, perception, cognition, communication, and behavior [1]. Epidemiologic studies indicate that the incidence of CP is approximately 2–3 per 1000 live births. Despite remarkable advances in obstetric and neonatal care, the overall prevalence of CP has changed little over the past several decades [2]. CP is a lifelong neurologic motor disorder and has a substantial impact on health care and social services costs, family welfare, and patient quality of life [3, 4]. Although known risk factors include prematurity, asphyxia, infection and inflammation, the causes of CP are unknown in most individuals [5, 6]. The pathogenesis and mechanism of CP have been studied and debated for more than 100 years, but the exact pathogenesis remains unclear. Many children with cerebral palsy do not have any known risk factor. Recently, families, twins, population-based, CNV and gene-association studies have strongly and directly suggested that genetic factors contribute to the etiology of CP [7–10].

CP is a complex disease. It is highly heterogeneous with regard to phenotype and etiology, and the different subtypes of CP might result from different causal pathways. Some cases may be caused by a single mutation in a single gene, but many may be caused by complex interactions of multiple genetic loci and environmental factors. However, the causes of CP are unknown in most individuals. An increasing association studies have explored the interactions between the susceptibility to CP and SNPs of candidate genes, including those involved in the inflammatory system and the coagulation cascade such as coagulation factor II/*V*/VII, lymphotoxin-alpha, tumor necrosis factor, IL-6, and IL-8, to determine whether they contribute to the causal pathway of CP [11–14].

Nitric oxide (NO) acts as a pleiotropic gaseous messenger molecule, and it is dynamically controlled during normal development and brain injuries. NO is synthesized from L-arginine by nitric oxide synthase enzymes (NOSs), of which there are three isozyme forms: neuronal NOS (nNOS, NOS1), inducible NOS (iNOS), and endothelial NOS (eNOS) [15]. The *NOS1* gene is the major isoform and is widely expressed throughout the brain, accounting for approximately 90% of NO in the CNS [16]. NO produced from NOS1 is involved in neurogenesis, neuronal differentiation and development [17, 18], and neuroprotection and neuropathology [19]. NO appears to play a critical role in hypoxia-ischemia (HI) brain injury which is a well-known risk factor for CP [20]. During hypoxic insults, excessive NO, which is produced by NOS1 in the cerebral tissue, produces toxic effects leading to neuronal death through several different mechanisms, such as the N -methyl- D –aspartic acid (NMDA) pathway. NMDA play a role in the pathology of multiple neurodegenerative diseases, such as Alzheimer’s, Parkinson’s and Multiple Sclerosis [21]. Recent studies have revealed that *NOS1* variants are associated with disorders such as Alzheimer’s disease [22], schizophrenia [23], and Parkinson’s disease [24]. The association of NOS1 with different diseases suggests a pleiotropic role of NOS1 in many physiological processes and a potentially shared pathomechanism.

Genetic and pharmacological studies have also provided valuable insights into the pathophysiology of NOS1. In a neonatal animal model of hypoxia-ischemia (HI), *NOS1* knockout mice were less vulnerable to HI-induced histopathological brain damage than their wild-type counterparts [25]. Selective inhibitors of NOS1, which reduce the NO concentration, were shown to dramatically improve the survival rate of fetal rabbits and ameliorate the symptoms of CP [26]. These results strongly suggest that NOS1 might be involved in the pathogenesis of CP. Although biological plausability for the involvement of NO in the pathogenesis of cerebral palsy is not clear. To our knowledge, no study has reported an association between the *NOS1* gene and CP. Therefore, we performed a comprehensive association study on polymorphisms of the *NOS1* gene and the risk of different subtypes of CP.

## Methods

### Study population

This was a case-control study based on a Chinese Han population. The study cohort included 652 CP patients and 636 healthy participants who were recruited from the centers for CP rehabilitation and Child Healthcare Departments in the Third Affiliated Hospital of Zhengzhou University, Zhengzhou Children’s Hospital and the First Affiliated Hospital of Henan Traditional Chinese Medical College. The CP group consisted of 198 female (30.4%) and 454 male (69.6%) patients ranging in age from 8 to 116 months. The control group was selected from healthy individuals who came to the facility for routine examinations and did not have neurologic conditions or predefined medical conditions; this group was comprised of 214 female (33.2%) and 422 male (66.8%) individuals ranging in age from 8 to 106 months. The diagnosis of CP was made by child neurologists, based on either a clinical examination or medical records and followed the guidelines proposed by the “Surveillance of CP in Europe” network [27]. Written informed consent was obtained from the parents or guardians on behalf of the infant participants. The study was approved by the Ethics Committee of Zhengzhou University.

The database of medical records contains information on the subtypes of CP, such as spastic; CP risk factors, such as preterm and birth asphyxia; symptoms concomitant with CP, such as mental retardation (MR); neonatal complications, such as periventricular leukomalacia (PVL) and hypoxic-ischemic encephalopathy (HIE); maternal factors, such as premature rupture of membrane (PROM); and neonatal encephalopathy (NE) (Table 1). The evaluation standards were consistent with those in our previous published study [13]. NE is a clinical syndrome of disordered neurological function, which includes not only HIE but also intracranial hemorrhage, hypoglycemia, severe hyperbilirubinemia, various metabolic disorders, neurodegenerative disorders, and intracranial infection, among other disorders [28].

**Table 1:** Clinical characteristics of all participants

Characteristic	CP cases (*n* = 652)	Controls (*n* = 636)
Sex (male:female)	454:198	422:214
Preterm (< 37 weeks)	37	9
< 2500 g	30	2
BIRTH Asphyxia	187	11
TYPE OF CP		
Spastic CP	438	–
CP with spastic tetraplegia	238	–
COMPLICATION		
CP with PVL	54	–
CP with HIE	91	–
CP with MR	248	–
CP with NE	261	–
MATERNAL FACTORS		
PROM	62	24
TPL	50	27
PIH	22	5

*CP* cerebral palsy, *PVL* periventricular leukomalacia, *HIE* hypoxic-ischemic encephalopathy, *MR* mental retardation, *NE* neonatal encephalopathy, *PROM* premature rupture of membrane, *TPL* threatened premature labor, *PIH* pregnancyinduced hypertensio

### SNP selection and genotyping

Altogether, six SNPs (rs3782219, rs6490121, rs2293054, rs10774909, rs3741475, and rs2682826) of the *NOS1* gene with minor allele frequencies in the Chinese Han population greater than 0.1 were selected from the dbSNP database (www.ncbi.nlm.nih.gov/SNP) and the phase II genotyping data of the HapMap project (http://www.1000genomes.org/). rs2293052 (exon 13) and rs3741475 (exon 22) were synonymous mutation, rs2682826 is located in 3’UTR, while the other three SNPs are located in intron: rs3782219 (intron 1), rs6490121 (intron 10), 10,774,909 (intron 20). Exception for rs10774909, the rest 5 SNPs were reported in published literature online, which were associated with disorders, such as Schizophrenia, Alzheimer’s disease, Parkinson’s disease.

Genomic DNA was extracted from whole blood of CP patients and controls, using the AxyPrep Blood Genomic DNA Miniprep Kit (Axygen Biosciences, Union City, CA, USA) according to a standard protocol. Probes and primers were designed using the SEQUENOM online tools (https:// http://www.sequenom.com), and the sequences are available upon request. After the amplification of polymorphism-spanning fragments by multiplex polymerase chain reaction (PCR), the selected SNPs were genotyped using the MassARRAY system (Sequenom, Inc., San Diego, CA) following the manufacturer’s directions (http://www.sequenom.com). SpectroTYPER software (Sequenom, Inc.) was used to automatically perform genotype calling with a set of digital filters optimized for the mass spectra of oligonucleotides. The individual who analyzed the genotype results was blinded to the clinical data.

### Statistical analysis

Hardy-Weinberg equilibrium testing was performed to analyze the allele and genotype frequencies, using the SHEsis online software platform (http://analysis.bio-x.cn/). Linkage disequilibrium was measured using standardized D’, and the discrepancies in allele and genotype frequencies at single loci between patients and controls were compared using a Monte Carlo simulation strategy. The number of observations for each haplotype were compared by χ2 tests. All reported *p* values are two-tailed, and statistical significance was set at *p* < 0.05. The relative risk was approximated by the estimate of odds ratio (OR), and for each OR, a 95% confidence interval (CI) was computed. All statistical analyses were performed using the Statistical Package for the Social Sciences (SPSS version 19.0) and GraphPad Prism 6.0 software package (version 6.0 for Windows, GraphPad, La Jolla, CA, USA). For all comparisons, multiple testing for each individual SNP was corrected using the SNPSpD program (http://gump.qimr.edu.au/general/daleN/matSpD/), which is based on the linkage disequilibrium. Multiple testing for each haplotype was corrected by the Bonferroni correction. Statistical efficacy was evaluated with G.power 3.1 software.

## Results

### Overall analysis

Power analysis showed that the current sample size had > 85% power for testing a significant association (ɑ < 0.05) when an effect size index of 0.1 was used. The genotypic distribution of the selected SNPs, except for rs6490121 (*p* = 0.0338), did not deviate from Hardy-Weinberg equilibrium (*p* > 0.05) among the control population (Table 2). Therefore, rs6490121 was excluded from further tests. For the other five SNPs, the genotype frequencies of rs2682826 (*p* = 0.046) were different between all CP patients and the controls, but the differences disappeared after SNPSpD correction (Table 2). There were no significant differences in the allele and genotype frequencies of rs3782219, rs6490121, rs3741475, rs10774909, and rs2293054 between CP cases and controls.

**Table 2:** Allele and genotype frequencies of SNPs in CP patients and controls

Group	Allele frequency		*P*	OR (95% CI)	Genotype frequency			*P*	*Hardy-Weinberg equilibrium test*
rs3782219	C	T			C/C		T/T		
Case	764 (0.586)	540 (0.414)	0.242	0.910	216 (0.331)		104 (0.160)	0.354	0.936
Control	774 (0.608)	498 (0.392)		(0.778~ 1.066)	235 (0.369)	0.939	97 (0.153)		
rs6490121	A	G			A/A		GG		
Case	831(0.637)	473(0.363)	0.888	0.989	268(0.411)		89(0.137)	0.585	0.0470
Control	814(0.640)	458 (0.360)		(0.842~ 1.1609)	272 (0.428)	0.047	94 (0.148)		
rs2293054	A	G			A/A		G/G		
Case	270 (0.207)	1034 (0.793)	0.607	1.052	29 (0.044)		411 (0.630)	0.877	0.204
Control	253 (0.199)	1019 (0.801)		(0.868~ 1.274)	26 (0.041)	0.204	409 (0.643)		
rs10774909	C	G			C/C		G/G		
Case	887 (0.680)	417 (0.320)	0.958	1.004463	293 (0.449)		58 (0.089)	0.496	0.107
Control	864 (0.679)	408 (0.321)		(0.8512~ 1.185)	294 (0.462)	0.107	66 (0.104)		
rs3741475	A	G			A/A		G/G		
Case	335 (0.257)	969 (0.743)	0.519	0.944	35 (0.054)		352 (0.540)	0.094	0.918
Control	341 (0.268)	931 (0.732)		(0.792~ 1.125)	52 (0.082)	0.918	347 (0.546)		
rs2682826	A	G			A/A		G/G		
Case	335 (0.257)	969 (0.743)	0.709	0.967	34 (0.052)		351 (0.538)	0.046	0.835
Control	335 (0.263)	937 (0.737)		(0.811~ 1.153)	52 (0.082)	0.835	353 (0.555)		

The SNP pairs rs3741475/rs2682826, rs3741475/rs10774909, and rs10774909/rs2682826 exhibited strong linkage disequilibrium (LD) (D’ > 0.9) (Additional file 1: Table S1). Haplotype analysis is a powerful strategy for resolving the controversy regarding association studies based on individual polymorphisms and determining whether the SNPs would have greater predictive value when analyzed together. We analyzed only the common haplotypes comprised of rs10774909, rs3741475, and rs2682826 (those with frequency < 0.01 were excluded from the analysis), but no statistically significant difference was found between all patients and controls (data not shown).

### Subgroup analysis

CP is highly heterogeneous with regard to clinical presentation, etiology, and pathogenesis. Subgroup analysis was performed to evaluate the potential relationship between genotypes and clinical features such as sex, gestational age, CP subtype, and neonatal complications (Tables 3, 4 and 5). The results of the subgroup analysis indicated significant differences in the allele or genotype frequencies in these CP subgroups, including male sex, birth asphyxia, spastic type, spastic tetraplegia, and NE. However, the associations of rs3741475 and rs2682826 with male CP, rs10774909 with CP + birth asphyxia, and rs2682826 with spastic CP disappeared after adjusting for multiple tests, using the program SNPSpD. There were no differences in allele or genotype frequencies in the other CP subgroups classified according to gestational age, birth weight, or fetal growth restriction (data not shown).

**Table 3:** Allele and genotype frequencies of SNPs in CP patients with spastic tetraplegia and controls

Group	Allele frequency		*P*	OR (95% CI)	Genotype frequency			*P*
rs3782219	C	T			C/C	C/T	T/T	
Case	255 (0.536)	221 (0.464)	0.006^a^	0.742	63 (0.265)	129 (0.542)	46 (0.193)	0.013
Control	774 (0.608)	498 (0.392)		(0.600~ 0.918)	235 (0.369)	304 (0.478)	97 (0.153)	
rs2293054	A	G			A/A	A/G	G/G	
Case	110 (0.231)	366 (0.769)	0.140	1.211	16 (0.067)	78 (0.328)	144 (0.605)	0.226
Control	253 (0.199)	1019 (0.801)		(0.939~ 1.560)	26 (0.041)	201 (0.316)	409 (0.643)	
rs10774909	C	G			C/C	C/G	G/G	
Case	328 (0.689)	148 (0.311)	0.695	1.047	106 (0.445)	116 (0.487)	16 (0.067)	0.159
Control	864 (0.679)	408 (0.321)		(0.834~ 1.313)	294 (0.462)	276 (0.434)	66 (0.104)	
rs3741475	A	G			A/A	A/G	G/G	
Case	108 (0.227)	368 (0.773)	0.079	0.801	8 (0.034)	92 (0.387)	138 (0.580)	0.043
Control	341 (0.268)	931 (0.732)		(0.625~ 1.027)	52 (0.082)	237 (0.373)	347 (0.546)	
rs2682826	A	G			A/A	A/G	G/G	
Case	109 (0.229)	367 (0.771)	0.142	0.831	8 (0.034)	93 (0.391)	137 (0.576)	0.042
Control	335 (0.263)	937 (0.737)		(0.649~ 1.064)	52 (0.082)	231 (0.363)	353 (0.555)	

^a^ After the SNPSpD correction, *p* = 0.023

**Table 4:** Haplotype analysis between patients with spastic tetraplegia and controls

Haplotype	case(frequency)	control(frequency)	*P*-value	OR(95% CI)
C G G	323.66 (0.680)	852.26 (0.670)	0.745	1.039 [0.826~ 1.306]
G A A	103.66 (0.218)	324.60 (0.255)	0.099	0.809 [0.629~ 1.041]
G GG	42.34 (0.089)	74.71 (0.059)	0.025*	1.561 [1.054~ 2.312]
Global result			0.034	

Abbreviations: *OR* odds ratio, *CI* confidence interval

Loci chosen for haplotype analysis: rs10774909, rs229305, rs2682826

Haplotype frequency < 0.01 in both control & case has been dropped

*After Bonferroni correction, *p* = 0.075

**Table 5:** Allele and genotype frequencies of SNPs in CP patients with neonatal encephalopathy and controls

Group	Allele frequency		*P*	OR (95% CI)	Genotype frequency			*P*
rs3782219	C	T			C/C	C/T	T/T	
Case	300 (0.575)	222 (0.425)	0.185	0.869	82 (0.314)	136 (0.521)	43 (0.165)	0.289
Control	774 (0.608)	498 (0.392)		(0.707~ 1.069)	235 (0.369)	304 (0.478)	97 (0.153)	
rs2293054	A	G			A/A	A/G	G/G	
Case	122 (0.234)	400 (0.766)	0.100	1.228	15 (0.057)	92 (0.352)	154 (0.590)	0.256
Control	253 (0.199)	1019 (0.801)		(0.961~ 1.560)	26 (0.041)	201 (0.316)	409 (0.643)	
rs10774909	C	G			C/C	C/G	G/G	
Case	343 (0.657)	179 (0.343)	0.364	0.905	98 (0.375)	147 (0.563)	16 (0.061)	**0.001** ^a^
Control	864 (0.679)	408 (0.321)		(0.729~ 1.123)	294 (0.462)	276 (0.434)	66 (0.104)	
rs3741475	A	G			A/A	A/G	G/G	
Case	145 (0.278)	377 (0.722)	0.675	1.050	10 (0.038)	125 (0.479)	126 (0.483)	**0.003** ^b^
Control	341 (0.268)	931 (0.732)		(0.836~ 1.319)	52 (0.082)	237 (0.373)	347 (0.546)	
rs2682826	A	G			A/A	A/G	G/G	
Case	145 (0.278)	377 (0.722)	0.531	1.076	9 (0.034)	127 (0.487)	125 (0.479)	**0.0005** ^c^
Control	335 (0.263)	937 (0.737)		(0.856~ 1.352)	52 (0.082)	231 (0.363)	353 (0.555)	

^a^After the SNPSpD correction, *p* = 0.004; ^b^After the SNPSpD correction, *p* = 0.012; ^c^After the SNPSpD correction, *p* = 0.002

Notably, the T allele of *NOS1* SNP rs3782219 was negatively associated with spastic quadriplegia (OR = 0.742, 95% CI = 0.600–0.918, after SNPSpD correction, *p* value = 0.023) (Table 3). The genotype frequencies of rs3782219 (*p* = 0.013), rs3741475 (*p* = 0.043), and rs2682826 (*p* = 0.042) also showed differences between spastic tetraplegia (*n* = 238) and controls, but the differences disappeared after SNPSpD correction. The haplotype analysis for rs10774909, rs3741475, and rs2682826 revealed a global *P*-value of 0.034 (Table 4). The haplotype “GGG” was found to be significantly associated with spastic tetraplegia (OR = 1.561, 95% CI = 1.054~ 2.312, *p* = 0.025), but the differences disappeared after Bonferroni correction (*p* = 0.075). There were significant differences in genotype between CP patients with NE (*n* = 261) and controls at rs10774909, rs3741475, and rs2682826 (*p* = 0.005, 0.015, and 0.003, respectively, after SNPSpD correction) (Table 5). The haplotype analysis of the three SNPs did not indicate significant differences between CP patients with NE and control subjects (data not shown).

## Discussion

The present study is the first to link a genetic variant with NOS1 gene to CP. We conducted a case-control study that included 652 CP patients and 636 healthy controls. Given that the sample size was sufficient for an appropriate statistical analysis, the likelihood of a type II error appears to be considerably low. Our result has shown that there was no significant association between *NOS1* and susceptibility to CP.

CP cases are highly heterogeneous with regard to both etiology and clinical phenotype, and different clinical phenotypes may have different pathogenesis [29]. Genetic factors might be associated with certain sub-types of CP [30]. According to the stratified analysis of factors such as sex, gestational age, birth weight, risk factors, clinical classification, complications and others. The T allele of *NOS1* SNP rs3782219 was negatively associated with spastic quadriplegia, and the genotype frequencies of rs10774909, rs37841475, and rs2682826 were significantly associated with CP + NE. Both spastic tetraplegia and CP + NE are serious forms of CP, suggesting that NOS1 likely plays a more important role in the pathogenesis of severe CP.

The etiology and pathogenesis of NE are not clear, and the known risk factors include HIE, intracranial hemorrhage, hypoglycemia, severe hyperbilirubinemia, a variety of metabolic disorders, neurodegenerative diseases, and intracranial infection [31]. Those also were risk factors for CP. In addition, NOS1 inhibitors can effectively reduce the degree of an ischemic brain injury. In view of the important role of NO in ischemic brain injuries, the association of the NOS1 gene with CP + NE also indirectly suggests that hypoxic brain damage may play an important role in the pathogenesis of CP.

NO is a highly diffusible gas that easily penetrates biological membranes and participates in a variety of important biological processes in the brain, such as immune responses, the release and delivery of neurotransmitters [32–34]. During intrauterine or hypoxic insults, excessive NO is produced by NOS1 in the cerebral tissue. Abnormal levels of NO have negative effects on the developing fetal brain through a wide range of mechanisms, such as glutamate and N-methyl-D-aspartate receptor (NMDAR) activation, resulting in excitotoxicity, oxidative stress, and inflammatory responses [35, 36]. It has been postulated that NMDA receptors play an essential role in the pathogenesis of CP [37, 38]. NMDA receptors are essential for excitatory synaptic transmission in the CNS. During in the process of hypoxic injury, excessive NO trigger the activity of NMDA receptors, leading to intracellular calcium ion influx, lipid peroxidation and free radical production. Those processes ultimately results in neuronal cell injury and irreversible brain damage. NMDA receptors were highly expressed in oligodendrocytes where glutamate toxicity could damage the myelin sheath that implies a role in synaptic stability and neuronal activity [39, 40].

The human *NOS1* gene is composed of 29 exons and 28 introns and maps to 12q24, which spans more than a 160 kb genomic region [41]. Moreover, its expression patterns are associated with the promoter-exon1 region [42]. Associations of *NOS1* with various diseases have been reported, such as schizophrenia, Parkinson’s disease, suicide, achalasia, multiple sclerosis, ischemic stroke, and hypertension. In previous studies found that the T allele of rs2293054 was associated with lower NIHSS scores and with NIHSS scores of ischemic stroke patients in different inherited model [43]. But in our result, rs2293054 showed no relationship with CP. To our knowledge only several studies are to investigate the relationship of rs3782219 SNP and rs3741475 with clinical diseases, it showed no relationship with disorders. However, we found the T allele of NOS1 SNP rs3782219 was negatively associated with spastic quadriplegia. The genotype frequencies of rs3741475 was significantly associated with CP + NE. Therefore, the T-allele of the rs3782219 and genotype of rs3741475 may contribute to the pathogenesis of clinical phenotypes in CP patients.

The synonymous SNP (rs2682826) located in the 3’-UTR of exon 29 of the *NOS1* gene was selected as the tag SNP for one of the most frequent haplotypes. The SNP rs2682826 is located close to several miRNA-binding sites in the gene’s 3′-UTR, and it likely affects the stability and translational efficiency of mRNA [43–45]. Some polymorphisms in NOS1 gene can directly affect the expression of mRNA, which change the levels of NO [46, 47]. While the majority of SNPs located in intron region do not affect the amino acid sequence, but they may be indirectly involved in the regulation of NOS1 expression or might be in linkage disequilibrium with a functional site. Rujescu et al. (2008) reported that the CGG haplotype which consists of 3 SNPs of NOS1 gene including rs2682826 SNP was significantly associated with suicide attempts [48]. The genotype frequencies of rs107749909, rs37841475, and rs2682826 were significantly associated with CP + NE, respectively. But the haplotype consisted of three highly linkage SNPs was no significantly associated with CP + NE. Future association studies with more systemic SNPs selection are thought to be needed to clarify the involvement of NOS1 in CP.

The etiology and pathogenesis of CP are complex. Regarding the genetic mechanism, CP may exhibit different genetic patterns, and the disease is polygenic in most CP cases. Although genome-wide association studies (GWASs) have been successful in identifying many cerebral disorder-associated loci, no GWAS on CP has been performed, and most of the CP-related genes have not been identified. Because of the complicated genetic architecture of CP, multiple genes might be involved in the etiology of CP, and the effect size of each individual risk allele is likely to be small. Moreover, the factors underlying inter-individual variations in the susceptibility to CP may also include demographic, clinical, and environmental variables. Our results need to be validated or replicated in other, larger population samples.

## Conclusions

There was no significant association between *NOS1* gene polymorphisms and CP at the total level, but *NOS1* was associated with spastic tetraplegia and CP + NE, suggesting that NOS1 may play a more important role in the pathogenesis of severe forms of CP. However, we only assessed five SNPs of the *NOS1* gene, and it is necessary to explore more *NOS1* gene variants. It is important to note that a comprehensive evaluation of the *NOS1* gene and CP requires a large sample for which sample independence can be validated and functional evidence is available. Furthermore, functional studies on the impact of these polymorphisms on gene expression in CP populations might help to define new therapeutic perspectives for CP.

## Additional file

Additional file 1: **Table S1.** Linkage disequilibrium among the five SNPs. The standardized D’ values are shown above the diagonal, and the r2 values are shown below the diagonal. (DOCX 12 kb)

## Abbreviations

CI: Confidence interval
CP: Cerebral palsy
HWE: Hardy-weinberg equilibrium
MAF: Minor allele frequency
NE: Neonatal encephalopathy
NOS1: Nitric oxide synthase 1
OR: Odds ratio
SNP: Single nucleotide polymorphism
