# Supplementary material for: Association of NOS1 gene polymorphisms with cerebral palsy in a Han Chinese population: a case-control study
Source: BMC Med Genomics. 2018 Jun 25;11:56. doi: 10.1186/s12920-018-0374-6 (PMC6019815; doi:10.1186/s12920-018-0374-6)
Supplement: Supplementary file 1 — Table S1. Linkage disequilibrium among the five SNPs. The standardized D’ values are shown above the diagonal, and the r2 values are shown below the diagonal. (DOCX 12 kb) [file 12920_2018_374_MOESM1_ESM.docx]

**Table S1. Linkage disequilibrium among the five SNPs**

| D'/ r2 | rs3782219 | rs2293054 | rs10774909 | rs3741475 | rs2682826 |
| --- | --- | --- | --- | --- | --- |
| rs3782219 |  | 0.414 | 0.083 | 0.045 | 0.050 |
| rs2293054 | 0.065 |  | 0.212 | 0.189 | 0.195 |
| rs10774909 | 0.005 | 0.024 |  | **0.946** | **0.948** |
| rs3741475 | 0.000 | 0.026 | 0.676 |  | **0.984** |
| rs2682826 | 0.001 | 0.028 | 0.670 | 0.956 |  |

The standardized D’ values are shown above the diagonal, and the r^2^ values are shown below the diagonal.
